# A comparison of patient dose and occupational eye dose to the operator and nursing staff during transcatheter cardiac and endovascular procedures

**DOI:** 10.1038/s41598-023-28704-y

**Published:** 2023-02-10

**Authors:** Kelly S. Wilson-Stewart, Davide Fontanarosa, Eva Malacova, Jamie V. Trapp

**Affiliations:** 1 School of Chemistry and Physics, Faculty of Science, Queensland University of Technology, 2 George Street, Brisbane, QLD 4000 Australia; 2 Centre for Biomedical Technologies, Queensland University of Technology, Kelvin Grove, Brisbane, QLD 4059 Australia; 3 Cardiovascular Suites, Greenslopes Private Hospital, Greenslopes, Brisbane, QLD 4120 Australia; 4 School of Clinical Sciences, Faculty of Health, Queensland University of Technology, 2 George Street, Brisbane, QLD 4000 Australia; 5 QIMR Berghofer Medical Research Institute, 300 Herston Road, Herston, Brisbane, QLD 2006 Australia

**Keywords:** Cardiac device therapy, Interventional cardiology, Cardiology, Health occupations, Biomedical engineering, Cardiovascular biology

## Abstract

The number and complexity of transcatheter procedures continue to increase, raising concerns regarding radiation exposure to patients and staff. Procedures such as transaortic valve implantations (TAVI) have led to cardiologists adopting higher dose techniques, such as digital subtraction angiography (DSA). This study compared the estimated patient and occupational eye dose during coronary angiography (CA), percutaneous coronary intervention (PCI), TAVI workups (TWU), TAVI, endovascular aneurysm repairs (EVAR), and other peripheral diagnostic (VD) and interventional (VI) vascular procedures. A quantitative analysis was performed on patient dose during 299 endovascular and 1498 cardiac procedures. Occupational dose was measured for the cardiologists (n = 24), vascular surgeons (n = 3), scrub (n = 32) and circulator nurses (n = 35). TAVI and EVAR were associated with the highest average dose for all staff, and significantly higher patient dose area product, probably attributable to the use of DSA. Scrub nurses were exposed to higher average doses than the operator and scout nurse during CA, VD and VI. Circulating nurses had the highest average levels of exposure during TAVI. This study has demonstrated that EVAR and TAVI have similar levels of occupational and patient dose, with a notable increase in circulator dose during TAVI. The use of DSA during cardiac procedures is associated with an increase in patient and staff dose, and cardiologists should evaluate whether DSA is necessary. Scrub nurses may be exposed to higher levels of occupational dose than the operator.

## Introduction

As fluoroscopically guided interventional procedures are being performed by an increasing number of medical specialities, procedures are being undertaken beyond their traditional location within the radiology department^1^. Recently, there have been notable advancements in catheter-mounted vascular devices and improvements in the quality of fluoroscopic imaging. While this presents the opportunity to treat complex vascular pathology less invasively, it also raises concerns over the increased radiation dose to the patients undergoing procedures and the staff performing them^2,3^.

Tissue damage due to radiation exposure can be categorized into stochastic and deterministic effects^4,5^. Radiation which induces the damage or death of a large population of cells is deterministic in nature and typically requires a threshold level of exposure to be reached before the biological effect manifests^5,6^. Incidences of skin effects in patients following x-ray guided transcatheter procedures have been widely reported^7,8^. Stochastic effects are thought to occur due to a random interaction which alters a single or small number of cells and may lead to the induction of malignancies or inheritable mutations^6,9^. Stochastic changes may occur after any exposure to radiation^10^. Due to the long latency period between exposure and the development of cancer, and the high prevalence in the general population, it is difficult to directly link medical exposure and oncogenesis^10^. There is increasing concern over the dramatic increase in the frequency of high-dose investigations that patients undergo and the potential cumulative impact of radiation exposure^2,11^.

The implications of radiation exposure are also of concern to staff. As with the potential stochastic effects on patients, direct relationships between occupational exposure and oncogenesis are difficult to prove^12,13^. There have been reports of a potential causal relationship between occupational exposure and the development of skin, breast and brain cancer^14–16^. There are also reports of DNA damage, chromosomal aberrations, genomic instability^17,18^ and cardiovascular damage at low levels of radiation exposure^19^. There is clear evidence of deterministic effects due to occupational exposure during fluoroscopically guided procedures. There are concerningly high levels of posterior subcapsular cataracts (PSC) reported among cardiology staff. One study demonstrates a 79% prevalence of PSC in occupationally exposed staff, contrasting to the 7.1% in an unexposed group^20^. The importance of investigating the occupational dose to the eye is needed not only to quantify dose levels but also to raise awareness and promote better radiation protection^21^.

Patient and operator dose during coronary angiography has been well researched^22–24^. Recent advances in catheter-mounted devices such as transaortic valve implantation (TAVI) have seen cardiologists utilize procedural imaging similar to endovascular angiography and employ tools such as digital subtraction angiography (DSA). The dose implications of this are less well represented in literature^25,26^. There is also a lack of literature comparing patient radiation exposure during cardiac and endovascular procedures^27^. While it has been stated that occupational exposure to vascular surgeons can match that of cardiologists^11^, there is very little published research comparing the specialities. Additional investigations measuring the dose levels for staff other than the operator are also needed^28,29^.

## Aim

This study compares the levels of patient, operator and nursing staff doses for diagnostic and interventional coronary angiography and intervention, TAVI, endovascular aneurysm repairs (EVAR) and peripheral vascular procedures.

## Materials and methods

Dose information during angiographic procedures was prospectively measured in three dedicated suites using Philips Allura Xper angiography equipment (Philips Healthcare, Best, Netherlands), conducted at a large tertiary hospital between February 2017 and August 2019. Data were collected for coronary angiography (CA), percutaneous coronary intervention (PCI), TAVI, TAVI workups (TWU), peripheral diagnostic (VD) and interventional (VI) vascular procedures, as well as EVAR. The PCI category included procedures that included a diagnostic coronary angiogram and intervention and stand-alone PCIs.

Patient air kerma (AK) and kerma area product (KAP) (also known as dose area product (DAP)) were retrieved from dose reports. Air Kerma (also referred to as incident, cumulative or reference air kerma) was measured at a reference point located 15 cm from the isocentre towards the tube.

The International Electrotechnical Commission have a regulatory limit which allows for a ± 35% deviation in the accuracy of the index reporting of AK and KAP^30,31^. Medical physicists performed annual testing to ensure compliance and calibration. The reported values have been obtained following the procedures detailed in "Accuracy and calibration of integrated radiation output indicators in diagnostic radiology: A report of the AAPM Imaging Physics Committee Task Group 190”^32^.

**Figure 1:**
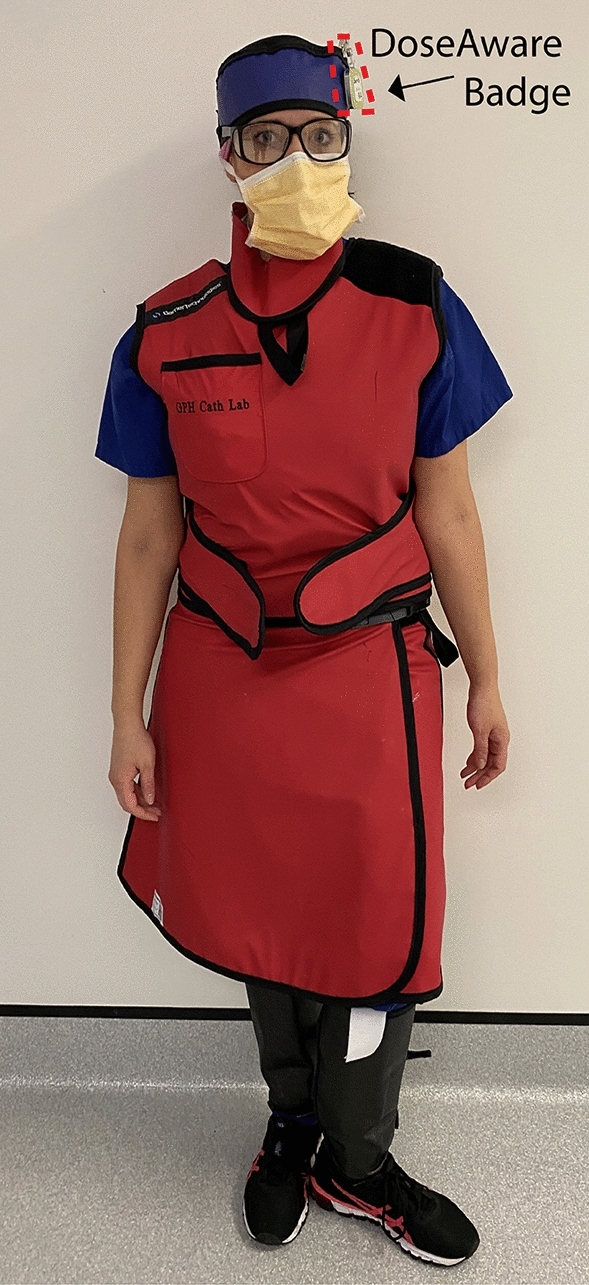
Typical personal lead shielding for scrub staff, including wrap-around style coat and skirt, thyroid shield, lead/lead equivalent skull cap and lead shin protectors. The DoseAware badge was mounted external to the protective equipment.

**Figure 2:**
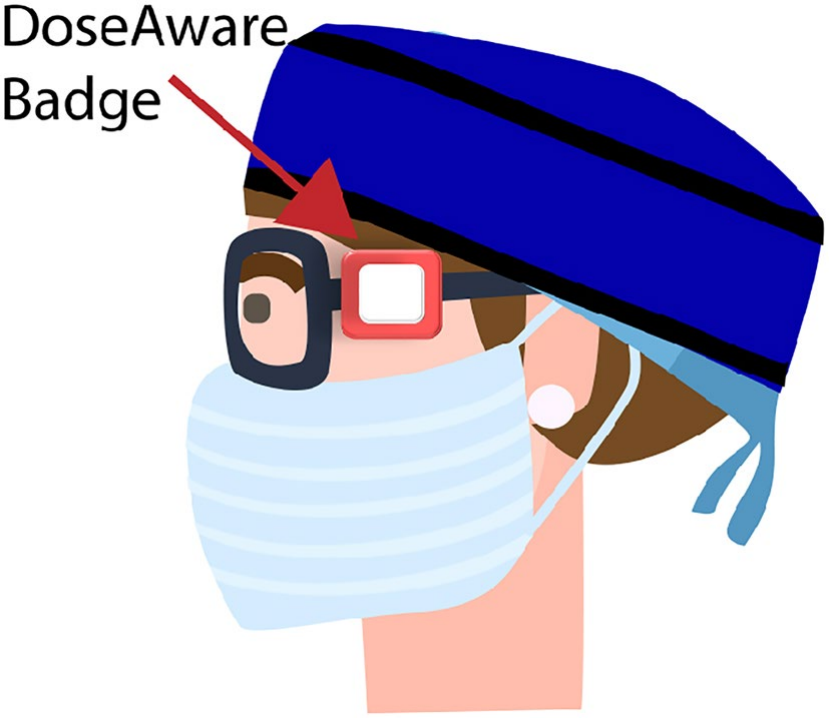
DoseAware badges were worn near the left eyeof staff, either attached to the arm of glasses, or to the lead/theatre cap.

Occupational dose to the cardiologist (n = 24), vascular surgeon (n = 3), scrub (n = 32) and circulator nurse (n = 35) was measured via a DoseAware dosimeter (Philips Healthcare, Best, Netherlands) worn near the left eye (Figs. 1 and 2). This site was chosen due to higher levels of scattered radiation to the eye nearest the x-ray tube^33–35^.

DoseAware badges consist of a solid-state active personal dosimeter which log the dose per second cumulatively^36^. Occupational dosimeter measurements will have a degree of variability due to the effect of photon incident angles, energy range and pulsed-field characteristics^37^. Detection by DoseAware badges has a reported uncertainty of 5%^38^ and has been demonstrated to detect satisfactorily within varying fields such as dose equivalent rate, peak high voltage and pulse width^39,40^. Prior to the commencement of the study, simultaneous dosimetric measurements were compared to a RaySafe X2 dosimeter (Raysafe, Sweden) to ensure the accuracy of the detection of scatter radiation. In addition, individual calibration certificates with traceability to the National Institute of Standards and Technology and Physikalisch-Technische Bundesanstalt were provided by the manufacturer^41^.

Dosimeters are calibrated to illustrate the equivalent dose at a certain depth. To provide an estimate of eye lens dose, ideally a Hp(3) dosimeter would be used to reflect the personal dose equivalent at 3 mm. Unfortunately, at the time of data collection, very few dedicated Hp(3) dosimeters were commercially available. Alternative operational quantities, such as Hp(0.07) or Hp(10), which are more widely available are sufficiently reliable for measuring exposure to the eye^42–44^, especially when worn in close proximity^45^. DoseAware badges with a calibration of Hp(10) when worn near the eye provide an appropriate measurement of eye dose, acknowledging a potential 5–15% overestimation^45^. DoseAware measures radiation cumulatively, and this information was downloaded every 14 days. Procedural commencement and conclusion times were noted so downloaded dosimetry data could be accurately assigned to the relevant case. Manufacturer specifications state that DoseAware has a detectable dose range 1 µSv–10 Sv^38^. The authors determined a detection rate down to 0.02 µSv, and it is noted that this range of validity cannot accurately be estimated, and hence the doses < 1 µSv may be subject to greater levels of uncertainty, and hence were reported as such.

A cine and fluoroscopy rate of 15 frames per second (fps) was used for cardiac procedures and 7.5 fps during fluoroscopy in endovascular cases. DSA acquisitions were taken at 6 fps for post-deployment EVAR to check for endoleaks, 3 fps for abdominal and pelvic imaging, and incrementally reduced to 0.5 fps for distal leg vessels. The imaging protocol for TWU procedures differed from PCI and CA as DSA imaging of the pelvic and femoral arteries was included to visualise TAVI catheter access route. All operators had at least 15 years of experience performing angiographic procedures, and the same pool of experienced nursing staff performed both cardiac and vascular procedures.

Staff wore thyroid shields and wrap-around lead skirts and tops, with additional shin protection if scrubbed (Fig. 1). It was also routine for scrub nurses and operators to wear lead glasses. The nursing staff utilized lead skull caps more often than the operators. DoseAware badges were worn near the left eye (near the temple) of staff, external to protective equipment (Figs. 1, 2 and 4). Figure 4 demonstrates routine positioning of the cardiologist/vascular surgeon and the scrub nurse during procedures.

**Figure 3:**
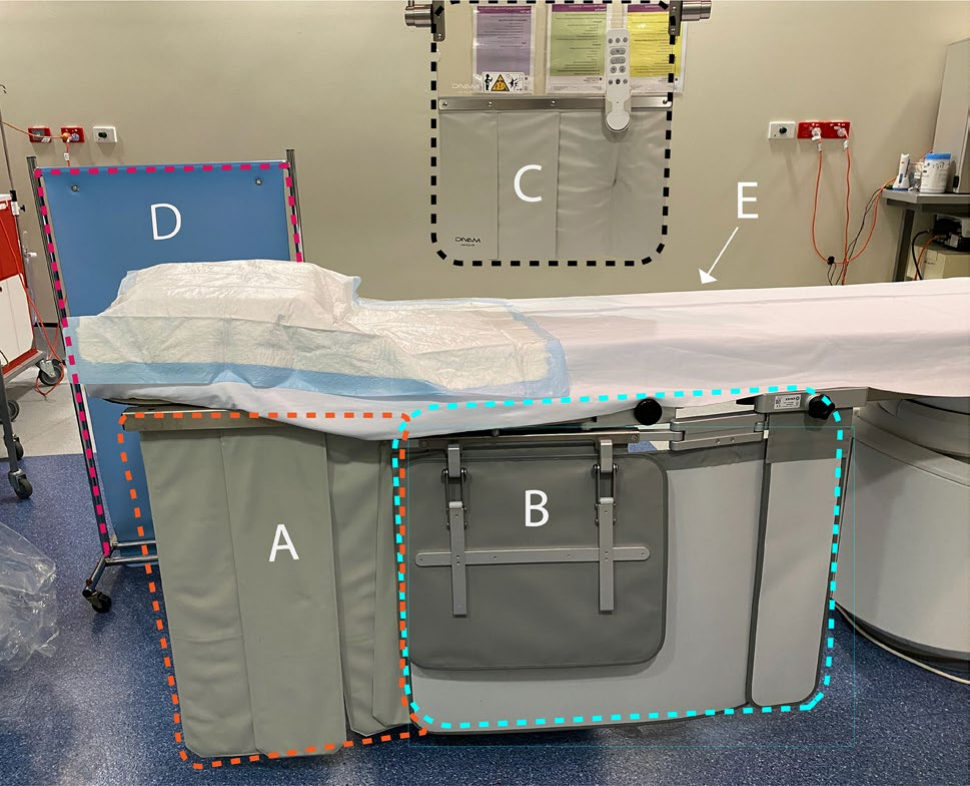
A common arrangement of lead shielding during procedures. (**A**) Single bank of table-mounted lead shielding to protect the lower body of the staff located beside the table; (**B**) Additional bank of lead shielding (not always utilized) to provide additional protection when the table is extended out from the table mount; (**C**) adjustable ceiling-mounted lead shield; (**D**) moveable lead shielding use by the circulating nurse; (**E**) Location of an additional bank of lead shielding on right side of the table, often employed during EVAR and TAVI, mirrors the position of B.

**Figure 4:**
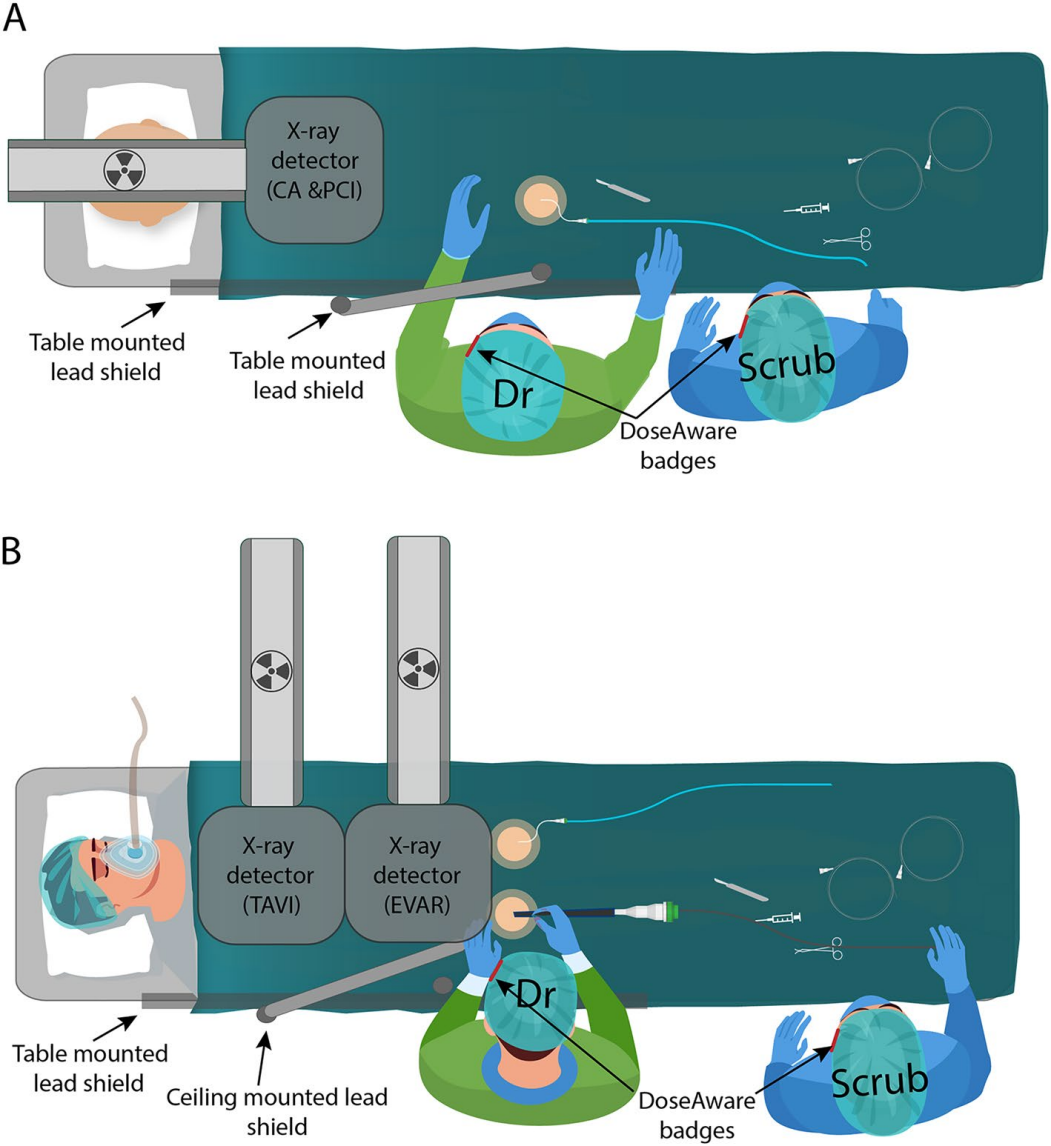
Demonstration of common staff positions in relation to the x-ray detector during CA and PCI (**A**), and TAVI and EVAR procedures (**B**). Location of the lead shielding and DoseAware badges are included. *Note—the x-ray tube is located under the patient table.

At least one bank of table-mounted lead shielding was present on the left side of the table, with additional shielding often utilized during EVAR procedures, as demonstrated in Fig. 3. Scrub nurses regularly used the moveable lead shield when located on the unshielded side of the table (Fig. 4).

Approval was granted by the Ramsay Health Care QLD Human Research Ethics Committee (Protocol number—16/67), and informed, written consent was obtained from staff participants. Research was conducted in accordance with the National Health and Medical Research Council guidelines. As all identifying information was removed prior to analysis, patient consent was deemed unnecessary by the ethics committee. Written informed consent was also obtained to publish identifying images in an online open-access publication. The research was conducted in accordance with the National Health and Medical Research Council guidelines.

### Statistical analysis

Eye doses to the cardiologist, scrub and circulator nurse were log-normally distributed based on normal quantile plots and thus needed to be log-transformed for the analyses. Other variables such as fluoroscopy time, AK, KAP were also approximately log-normally distributed. All results of log-transformed variables were reported as geometric means with 95% confidence intervals (CIs). Means of log-transformed variables were exponentiated to obtain geometric means. This method of analysis was chosen due to its to its superiority in providing information on the magnitude of the effect being investigated, as opposed to p-values. In addition, a Pearson correlation was used to assess the degree of correlation between staff and patient dose and the relationship between patient BMI and patient dose. STATA version 15.1 (Stata Corporation, College Station, Texas USA) and Statistical Discovery Software JMP Pro (Version 15.2.0 SAS Institute, Cary, NC, USA) were used for all analyses.

## Results

Patient dose data (n = 1797) and occupational dose levels were collected for CA (n = 906), PCI (n = 548), TAVI (n = 21), TWU (n = 23), VD (n = 75), VI (n = 187), and EVAR (n = 37) procedures, as demonstrated in Table 1. Other procedural parameters are presented in Table 2. Any statements regarding occupational dose refers to the dose measured at the eye closest to the x-ray tube.

**Table 1:** Geometric means (95% CI) of staff and patient dose measurements for differing types of coronary and vascular procedures.

Staff dose (µSv)	Cardiac procedures					Vascular procedures			
	CA	PCI	TWU	TAVI	Total Cardiac	VD	VI (non-EVAR)	EVAR	Total vascular
Operator	< 1	< 1	3 (1, 6)	7 (0, 186)	1 (1, 2)	< 1	1	8 (4, 13)	< 1
n	728	427	20	1	1176	43	123	31	197
Scrub	1	< 1	2 (1, 4.)	3 (1, 7)	1 (1, 2)	< 1	1 (1, 2)	3 (2, 6)	1
n	684	358	16	19	1077	61	140	32	233
Circulator	< 1	< 1	< 1	< 1	< 1	< 1	< 1	< 1	< 1
n	408	297	14	13	732	38	93	23	154

Patient dose	Cardiac procedures					Vascular procedures			
KAP (Gy·cm^2^)	18 (17, 19)	32 (30, 35)	43 (30, 62)	93 (63, 138)	25 (22, 28)	20 (17, 25)	20 (18, 23)	110 (83, 147)	23 (22, 24)
AK (Gy)	0.32 (0.30, 0.34)	0.79 (0.73, 0.85)	0.44 (0.31, 0.63)	0.67 (0.46, 0.97)	0.13 (0.12, 0.15)	0.09 (0.08, 0.11)	0.12 (0.10, 0.13)	0.51 (0.38, 0.67)	0.45 (0.43, 0.48)
N	906	548	23	21	1498	75	187	37	299

AK—Air kerma; CA—coronary angiography; CI—confidence interval; KAP—kerma area product; EVAR—endovascular aneurysm repair; Gy—Gray; Gy·cm^2^—Gray centimeter squared; PCI—percutaneous coronary angiography; TAVI—transcatheter aortic valve implantation; TWU—TAVI workup; VD—vascular diagnostic; VI—vascular intervention; µSv—microSievert.

**Table 2:** Geometric means (95% CI) of fluoro time, cine runs and mean (95% CI) of patient BMI for differing types of coronary and vascular procedures.

	Fluoro time (mins)	Cine runs	Patient BMI
CA	3.05 (2.92, 3.18)	9.54 (9.31, 9.78)	29.75 (29.38, 30.11)
PCI	11.33 (10.72, 11.98)	21.36 (20.68, 22.05)	29.55 (29.08, 30.02)
TWU	6.68 (5.09, 8.77)	12.01 (10.27, 14.04)	27.38 (25.04, 29.72)
TAVI	18.27 (13.75, 24.29)	13.10 (11.12, 15.43)	29.77 (27.32, 32.22)
VD	1.66 (1.43, 1.93)	6.31 (5.79, 6.89)	28.50 (27.23, 29.77)
VI (non-EVAR)	5.50 (5.01, 6.04)	12.58 (11.90, 13.29)	28.27 (27.47, 29.08)
EVAR	14.37 (11.64, 17.73)	16.86 (14.90, 19.08)	28.14 (26.34, 29.95)

CA—Coronary angiography; BMI—body mass index; CI—confidence interval; EVAR—endovascular aneurysm repair; PCI—percutaneous coronary angiography; mins—minutes; TAVI—transcatheter aortic valve implantation; TWU—TAVI workup; VD—vascular diagnostic; VI—vascular intervention.

TAVI and EVAR were associated with the highest average dose for all staff (Table 1). EVARs resulted in the highest mean dose to the vascular surgeons (8 µSv), while TAVI had the highest mean dose for the cardiologist (7 µSv), but due to the low sample number, the 95% CI were too wide to reach significance when compared to other procedures. Operator dose during TWU (3 µSv) and EVAR (8 µSv) procedures were associated with a significant increase compared to CA, PCI, VI and VD, which were all < 1 µSv. The scrub nurse was exposed to significantly higher eye dose during TAVI (3 µSv) compared with CA (1 µSv), PCI and VD (< 1 µSv). The scrub nurse also had higher mean dose levels compared to other staff (operator and circulator) during CA (1 µSv), VD (< 1 µSv) and VI (1 µSv) PCI, TAVI, EVAR and VI were associated with a significantly higher dose to the circulator nurse when compared to CA (Table 1).

Average patient KAP was significantly higher during TAVI and EVAR when compared to all other procedural categories (Table 1). PCI had the highest mean AK (0.79 Gy) and were associated with significantly higher AK than CA (0.32 Gy), TWU (0.44 Gy), VD (0.09 Gy), VI (0.12 Gy) and EVAR (0.51 Gy). The fluoroscopy times during TAVI and EVAR were significantly longer than those for other procedures, with the exception of EVAR compared to PCI, which did not reach significance (Table 2). There was no significant difference of patient BMI across different procedural categories. PCI were also associated with a significantly higher number of cine runs than other procedural categories.

Table 3 shows the correlation of patient AK and KAP with staff dose during the different categories of procedures, as well as the correlation of patient BMI with patient AK and KAP. The dose to the scrub nurse was found to be highly correlated to patient AK during TAVI and EVAR. There was also a high positive correlation between scrub nurse dose and KAP during EVAR. Patient BMI had low correlation with patient dose during VD and VI procedures.

**Table 3:** Correlation coefficients for staff and patient dose.

Correlation	Cardiac procedures				Vascular procedures		
	CA	PCI	TWU	TAVI	VD	VI (non-EVAR)	EVAR
Operator/AK	0.3500	0.4419	0.4057	–	0.3625	0.4772	0.4750
Operator/KAP	0.3872	0.4936	0.2904	–	0.2920	0.5763	0.5883
Scrub/AK	0.2654	0.3157	0.4932	**0.7817**	0.3593	0.5564	0.7167
Scrub/KAP	0.2856	0.4483	0.5581	0.6571	0.2732	0.6285	**0.7737**
Circulator/AK	0.2389	0.3302	− 0.3229^	0.1359	0.2474	0.3423	0.6795
Circulator/KAP	0.2433	0.3445	− 0.1392^	0.0679	0.2581	0.3014	0.6138
Patient BMI/AK	0.4086	0.3905	0.6021	0.4959	0.1565	0.0691	0.6330
Patient BMI/KAP	0.3461	0.4067	0.6580	0.3120	0.1647	0.0697	**0.7550**

AK—Air kerma; BMI—body mass index; CA—coronary angiography; KAP—kerma area product; EVAR—endovascular aneurysm repair; PCI—percutaneous coronary angiography; TAVI—transcatheter aortic valve implantation; TWU—TAVI workup; VD—vascular diagnostic; VI—vascular intervention; only one data point available.

Values in bold indicate variables which indicate a strong correlation (> 0.75).

^Results indicate that as patient exposure increases, the occupational dose to the circulator nurse decreases.

## Discussion

With the number and complexity of cardiovascular imaging procedures increasing over the last decade, reducing radiation exposure to the patient and the staff has become a major challenge for modern imaging departments^1,46^. Occupational and patient dose comparisons during CA and PCI have been previously reported^47,48^. There is less literature comparing occupational and patient dose during coronary angiography and intervention to more recently adopted procedures such as TAVI. Comparisons of doses during cardiac and endovascular angiography, notably research investigating radiation exposure levels to the nursing staff, are also lacking. Existing literature measuring operator and scrub nurse (or personnel occupying a similar location) dose during femorally accessed TAVI and EVAR procedures is demonstrated in Table 4.

**Table 4:** Mean operator and scrub nurse dose during transfemoral EVAR and TAVI.

Author	Year published	Procedures	n	Imaging system	Operator dose (μSv)	Dosimeter position	Scrub Nurse^b^ dose (μSv)	Dosimeter position	Patient KAP (Gy∙ cm^2^)	Patient AK (mGy)
EVAR										
Kloeze et al.^49^	2014	EVAR-without RAD	18	Axiom Artis, Siemens	167.7	ULC	42.3	ULC	94.58	
					470.3	Left ring finger				
		EVAR-with RAD	18		73.0	ULC	21.4		86.38	
					236.8	Left ring finger				
Sailer et al.^50^	2015	FEVAR, TEVAR, EVAR	44	Xper with ClarityIQ Technology	170 (110^c^)	ULC	42	ULC	130 (116^c^)	
Kirkwood et al.^51^	2015	EVAR	13	Allura Xper FD20	6	ULC	3			
Timaran et al.^52^	2021	EVAR-standard magnification^a^	123	Philips Allura XperFD20	266	ULC	7	ULC		2.46
		EVAR—digital zooming^a^	28	Philips Allura XperFD20	101	ULC	3	ULC		1.38
This study		EVAR	31	Philips Allura XperFD20	7.6	Left eye	3.3	Left eye	110.60	0.51
TAVI										
Sánchez et al.^53^	2020	TAVI	33 (Operator) 19 (Scrub)	Philips Allura Clarity	10	ULC	1	ULC	44	
Sauren et al.^54^	2011	TAVI	11	Philips Allura Xper FD20	3^d^	ULC/Thyroid shield	2^b,d^	ULC/Hand/Feet	8840	0.53
Shatila^26^	2015	TAVI	21	Philips Allura XperFD20	101^e^	ULC	33^b,e^	ULC		
This study		TAVI	1 (Operator) 19 (Scrub)	Philips Allura XperFD20	7.27	Left eye	3.02	Left eye	93.33	0.67

Demonstrates the mean occupational doses to operator and scrub nurse (and patient dose, where provided) during transfemoral EVAR and TAVI, noting that only studies with similar staff location for nursing/other staff included.

AK—Air kerma; CA—coronary angiography; CI—confidence interval; KAP—kerma area product; EVAR—endovascular aneurysm repair; FEVAR—fenestrated endovascular aneurysm repair; Gy—Gray; PCI—percutaneous coronary angiography; mGy·cm^2^—milli Gray centimeter squared; RAD—radiation-absorbing drapes; TAVI—transcatheter aortic valve implantation; TEVAR—thoracic endovascular aneurysm repair; TWU—TAVI workup; VD—vascular diagnostic; VI—vascular intervention; ULC—upper left chest; µSv—microSievert; a—fenestrated and branched devices included; b—or similar position to the scrub nurse in this study; c—EVAR only; d—effective dose with a conversion factor of 5; e—median dose.

Noting that this study measured dose at the level of the eye, as opposed to the level of the upper left chest, the occupational doses in this study were similar to that reported by Kirkwood et al. (EVAR) and Sánchez et al. (TAVI) but much lower than other comparable studies^49,50,52^. This may indicate a degree of awareness amongst the participants of the current study regarding appropriate radiation protection measures.

Unsurprisingly, the average dose to the circulator nurse was significantly lower than other staff during CA, PCI, TWU, VD and VI, which can be explained by the ability of the circulator to move away from the area of greatest radiation scatter during procedures. This may also explain the negative correlation coefficients demonstrated in Table 3. The correlation between patient dose and circulator dose is also noted (Table 3) and this is hypothesized to be due to the surgical equipment transferred by the circulator nurse to the scrub nurse in close proximity to the patient, as well as the large irradiated area of the abdomen required during EVARs which will increase the x-ray scatter. The scrub nurse dose was associated with significantly higher radiation levels during CA compared to PCI. This was most likely due to the higher scrub nurse dose during diagnostic angiograms performed by diagnosticians (1 µSv), as opposed to those performed by interventionalists (< 1 µSv) (data not shown). Concerningly, it was demonstrated that scrub nurse dose was higher than the operator during CA and had a higher mean dose when considering the whole cardiac dataset. This is thought to be due to the high levels of radiation awareness within the department, and the operator’s diligent positioning of the ceiling-mounted lead shield, providing protection to the operator but not the scrub nurse. The majority of the literature to date identifies that operators are exposed to the highest levels of scatter radiation during procedures^50,52,53^. There is a small number of studies indicating that nurses can be exposed to greater doses than the operator^41,55^, and this perhaps provides an opportunity to reconsider the broadly held assumptions regarding occupational dose within a general sense, and investigate dose to staff in the local setting, so a bespoke approach to radiation protection can be implemented. Additionally, it should be noted that the occupational doses measured in this study, when extrapolated, fell well below the current International Commission on Radiological Protection’s (ICRP) recommended eye dose limitations and were low when evaluated against most comparable studies (Table 4).

It would be ideal if a dosimeter with an operational quantity of Hp(3) be worn as close as possible to the eye to provide an accurate estimation of lens dose. Currently, there is limited access to and affordability of dedicated eye dosimeters. It is also acknowledged that placement of the easily accessible personal dosimeters is distracting and impractical for the staff member. Alternative solutions have been investigated with Omar et al. calculating a formalism and estimation of the dose conversion factor for a dosimeter calibrated at Hp(10) worn at the chest to estimate eye lens dose. The results indicate (conservatively) that operator dose measured at the eye level is estimated to be double that measured at the chest level. It was also determined that the dose at eye level to the second scrubbed staff member (circulator nurse or assistant operator) is equivalent to the dose measured at the chest level^56^. There was no significant difference found between the patient BMI across the categories considered in this study, but it is recognised that the dose to the staff and patients during fluoroscopically guided coronary procedures is influenced by patient BMI^55^. Due to the complexity of anatomy, x-ray tube angulation is frequently utilized to provide effective imaging during cardiac and vascular procedures, and this has been demonstrated to greatly affect patient and staff dose^57^. Steeper angles result in higher doses to the patient due to the additional thickness of tissue for the beam to traverse^58^. As a result, occupational dose is also increased, especially when the under-table x-ray tube is rotated to a position where the scatter profile is close to the location of the staff^59^. Given the variability of the use of x-ray tube angulation in the clinical setting and the resulting effect this has on dosimeter measurements, investigating the effect of tube angle was beyond the scope of this investigation and would be better suited to a phantom study. We also note that there is a large discrepancy regarding the use of/positioning of radiation protection and the height of the staff, which has been demonstrated to affect occupational dose^60^.

The number of cine acquisitions was significantly higher in PCI than in other categories. Patient KAP and AK were also significantly higher during PCI than CA, VD and VI. AK was significantly higher during PCI than CA, TWU, EVAR, VD and VI. This is likely due to the greater use of magnification during coronary intervention than the other procedural categories, and this provides an indication of the potential for deterministic tissue effects post-procedure. Conversely, EVAR and TAVI were associated with significantly higher KAP than other categories. KAP is a measurement that reflects the total amount of radiation delivered to a patient, and hence it is understandable that this value is higher for TAVI and EVAR, given the greater volume (and thickness) of tissue irradiated.

DSA imaging has been shown to significantly increase the dose burden to patients^58^. It was anticipated that DSA would also contribute to higher radiation levels for the operators and nursing staff. This appears to be reflected in the average dose to all staff being higher during EVAR, TAVI and TWU (excluding circulator during TWU). It is noteworthy that the highest average doses to scrub nurses were received during EVAR and TAVI. Predictably, TAVI and EVAR also had longer average fluoroscopic times, with TAVI being significantly longer than CA, PCI, TWU, VD and VI. Authors have noted that fluoroscopy may be the largest contributor to KAP, followed by DSA^61^. This may also explain the low correlation between patient BMI and VI and VD procedures due to the lower fluoroscopy times.

Additionally, the dose to the staff is known to be correlated with patient dose^62^, and the length of fluoroscopic activation will affect occupational exposure levels^11^. This study has demonstrated that patient KAP and the radiation dose to the operator and scrub nurse are higher during TWU than CA and PCI, clearly indicating that the use of DSA is associated with increased patient and staff dose. This theory is also supported by the high correlation found between scrub nurse dose and patient KAP (0.77) and AK (0.72) during EVAR, and patient AK (0.78) during TAVI. Imaging of the femoral access route should be a component of preoperative planning in both EVAR and femorally accessed TAVI. Increased staff and patient dose due to the use of DSA can be mitigated by utilizing processing software post computed tomography angiography or magnetic resonance imaging to assess vascular anatomy and pathology on the approach route, as well as to determine appropriate tube angulations and landing zones^63^. Reports indicate that DSA does not improve diagnostic capabilities when imaging the femoral access site during coronary angiography^58^, and consideration should be given to avoiding pelvic artery DSA during TWU. To further reduce dose to the patient and staff, consideration could also be given to the use of contrast-enhanced ultrasound imaging to assess endoleaks post-EVAR deployment or the use of fusion imaging^64,65^.

### Limitations

The main limitation is the single-centre design which may render results less generalizable to other settings. Another one is the inclusion of only a single measurement for the operator dose during TAVI. Unfortunately, after the data collection period, it was discovered that some of the dose data were unusable. Additionally, the TAVI procedures were no longer being performed on the Philips equipment included in the investigation, having moved to a Hybrid theatre (Siemens), so comparable data could not be collected. It is worth noting that the single valid measurement, although not statistically relevant, was congruous with similar studies with dose levels consistent with the ones reported here^53,66^.

Occupational and patient radiation dose during fluoroscopically guided cardiovascular procedures is affected by many factors, including tube angulation, collimation and magnification, which were not reported in this study. This was due to the constant alteration of these factors throughout procedures within a clinical setting, and as such, these factors are better suited to a phantom investigation. It is acknowledged that there are uncertainties associated with risk projection when utilising the values of KAP and AK as dose metrics. While KAP and AK have the potential to provide an accurate reflection of organ dose or peak skin dose, in the clinical scenario they may provide a rough estimation only. An additional limitation is that the effect of individual staff members on occupational and patient dose was not evaluated.

## Conclusion

Exposure to ionizing radiation may have biological consequences. Given the potential implications of radiation exposure to both patients and staff, there is a need to keep exposure as low as reasonably achievable. To effectively accomplish this, a knowledge of the variables that influence occupational dose is essential. This study has demonstrated that EVAR and TAVI have similar levels of occupational and patient dose, with the notable increase in circulator dose during TAVI. The use of DSA during cardiac procedures is associated with an increase in patient and staff dose. Cardiologists need to consider if there is a clinical advantage to employing DSA imaging of the pelvic arteries during TAVI and TWU, especially if advanced imaging has been conducted via other modalities. Scrub nurses should be aware that their exposure levels may be higher than that of the operator, and ensure that they implement techniques to minimize personal dose. Additionally, staff should be mindful of their location during procedures and take opportunities to step away, such as using remote triggering of injectors, or standing behind additional shielding when close to the patient, such as controlling pacing during TAVI.

## Data Availability

The datasets generated during and/or analyzed during the current study are available in the QUT Research Data Finder, can be access via QUT—Research Data Finder.
